# Measurement of self-reported, facility-based labour and birth experiences: The Perinatal Experience Assessment Tool (PEAT)

**DOI:** 10.7189/jogh.12.04103

**Published:** 2022-12-29

**Authors:** Cathryn Ellis, Charles P Larson, Frank Bicaba, Abel Bicaba, An Nguyen, Jean Ramdé, Alexandra Otis

**Affiliations:** 1 Midwifery Program, Department of Family Practice, Faculty of Medicine, University of British Columbia, Vancouver, British Columbia, Canada; 2 Canadian Coalition for Global Health Research, Ottawa, Ontario, Canada; 3 Department of Epidemiology, Biostatistics and Occupational Health, School of Population and Global Health, Faculty of Medicine and Health Sciences, McGill University, Montréal, Québec, Canada; 4 Société d'Études et de Recherche en Santé Publique, Ouagadougou, Burkina Faso; 5 HealthBridge Vietnam, Hanoi, Vietnam; 6 Direction en santé mondiale, Faculté de Médecine, Université Laval, Québec City, Québec, Canada

## Abstract

**Background:**

Women and their families make decisions on accessing perinatal care based on their experiences in the health care system and on the experience of others around them. Receiving supportive maternity care which demonstrates respect is an essential part of quality care. Globally, and in low- and middle-income countries in particular, women report receiving mistreatment and a lack of respect during labour, childbirth and the early postnatal period. These experiences, if negative, may influence choices around place of birth, thus hindering the scale-up of facility-based births.

**Methods:**

We conducted a focussed review of the literature between 2010 and 2019 to identify recent research addressing the assessment of women’s experiences during childbirth in low- and middle-income country facilities. The World Health Organization (WHO) and White Ribbon Alliance themes and concepts of respectful maternity care served as a guide. Themes included disrespectful or abusive experiences such as verbal abuse or rudeness, abandonment, corruption, lack of privacy, failure to respect traditional practices, discrimination, and physical or sexual abuse. Experienced midwives in two low-resource countries contributed to the identification of appropriate indicators of respectful, non-abusive care, and eventual agreement as to which to include in an assessment tool monitoring women’s experiences.

**Results:**

Our review of the literature identified 18 publications meeting pre-established criteria. This resulted in the eventual selection of 33 indicators of respectful care sub-grouped under 9 domains: 1) communication/verbal interaction, 2) supportive care, 3) physical abuse, 4) non-consented care, 5) non-confidential care/lack of privacy, 6) stigma and discrimination, 7) abandonment/neglect, 8) detention/inability to pay, and 9) health facility conditions. We converted these indicators into questions to be asked by an interviewer during a short interview following discharge to assess the childbirth experience.

**Conclusions:**

The Perinatal Experience Assessment Tool (PEAT) may be used to monitor or evaluate the experiences that women report after facility-based childbirth. It can be administered by trained, independent interviewers in the facility following discharge. The PEAT enables maternity leaders to assess the extent to which maternity services are conducted in a respectful, non-abusive manner and modify practices and procedures as feasible and appropriate.

Patient experience is recognised to be one of the three pillars of quality in health care alongside clinical effectiveness and patient safety [1]. Broadly defined, patient experience encompasses effective communication, respect and dignity, and emotional support as experienced within a population’s health system [2]. In reference to facility-based childbirths, the World Health Organization (WHO) and its member states have declared “every woman has the right to the highest attainable standard of health, which includes the right to dignified, respectful health care” [3]. This has been supported by the White Ribbon Alliance that has focussed on the universal rights of childbearing women, emphasizing respectful care in childbirth [4]. Respect is not always present, as has been documented throughout all societies, but most importantly within resource-constrained settings serving women and newborns most vulnerable to preventable perinatal mortality and mortality [5]. Aside from the inherent interpersonal values in treating expectant women with dignity and respect, these self-reported experiences have been demonstrated to influence patient safety and clinical outcomes [6].

Disrespect during childbirth is a global problem [3]. The WHO has called on governments and development partners to carry out further research defining and measuring disrespect and abuse in health facilities worldwide [3]. Disrespectful or abusive experiences include verbal abuse or rudeness, abandonment, corruption, lack of privacy, failure to respect traditional practices, discrimination, and physical or sexual abuse [6-14]. These behaviours reflect provider heavy workloads, facility cultures, prejudices, privilege, and feelings of superiority [13]. A mixed-method systematic review offers a typology of mistreatment of women in facilities at different levels including various types of abuses, poor interactions between health care providers and users, failure to meet standards of care, and health system constraints [15].

Based upon postpartum interviews of women who had recently given birth, disrespectful and abusive care during labour and birth have been documented to commonly occur in lower income, resource-constrained settings. In Kenya [16] and Tanzania [17] this occurrence has ranged from 21 to 27% respectively. In a multi-country study carried out in Nigeria, Ghana, Guinea and Myanmar, overall between 10.7 and 30.7% of women in their early postpartum period reported physical or verbal abuse [18]. A systematic review of 12 studies in Nigeria reported such behaviours to range from 11 to 71% of facility-based childbirths [10]. Similarly, in a systematic review in India disrespect and abuse during childbirth varied between 10 and 77% [14]. In response there has been a call for health systems to measure and report health service user experiences [19].

This paper describes the development of a tool, the Perinatal Experience Assessment Tool (PEAT), intended to measure experiences with facility-based childbirth of women who recently gave birth in low-or middle-income countries. The PEAT is currently available in English and French. It is intended that the PEAT will assist health facilities in monitoring the experiential quality of care they are providing.

## METHODS

In 2019 Global Affairs Canada (GAC) funded the Canadian Partnership for Women’s and Children’s Health (CanWaCH) to establish a collaborative of research laboratories that would address the harmonization of specific health metrics to generate solutions to urgent data challenges in global health and gender equality [20]. Following acceptance of a protocol to identify priority health metrics addressing women and children’s health themes, a survey and interview of academic and leaders of the non-governmental organisations indicated that a tool to focus on patient-provider interaction was needed.

In order to develop this tool, our research team commenced with an initial literature review of studies carried out in low-and middle-income countries. The review was further refined to specifically address patient provider interactions during labour and birth. A focused literature review was carried out through searches in PubMed Central and Google Scholar. The search terms included respectful or disrespectful and or abusive maternity or labour and delivery care, patient-provider interaction during childbirth.

Inclusion criteria for the publication included: 1) peer-reviewed, 2) published from 2010 to 2019, 3) written in English or French, and 4) inclusion of a measurement tool or questionnaire addressing perinatal care in a low- or middle-income country setting. Within the identified articles, indicators of the perinatal experience that could be obtained from a post-partum interview were extracted. We assessed each indicator for relevance to a mother’s experience during labour and delivery and feasibility within a resource-constrained setting. The eventual wording of each indicator, expressed as a question, was subjectively agreed upon and in instances where several publications were cited for an indicator CE, CL and AO reached agreement for a representative wording. The indicators were then listed under domains consistently found in the articles reviewed (CL, AO). The resulting list of indicators and domains was then shared with four midwives who work in, and have had extensive experience in, low-income countries. Two of these midwives work in low-resource countries in settings where the survey is intended to be used. The aim of these consultations was to assess content validity and to reduce the indicators to a manageable number in the context of an exit interview, anticipating interviewers would likely be limited to 15 to 20 minutes per mother. In addition, the indicators were reviewed for relevance and redundancies by two co-investigators (CL and CE). Where agreement to omit was reached by at least five out of the seven reviewers the item was dropped and a final draft tool prepared. The remaining indicators were then externally reviewed by three members of the CanWaCH Health Metrics working group with monitoring and evaluation expertise. We asked reviewers to judge and comment on the content and feasibility of the PEAT as well as add or omit specific indicators. This led to a revision in the wording of some indicators, however, none were rejected and no additional items were suggested.

## RESULTS

**Figure 1:**
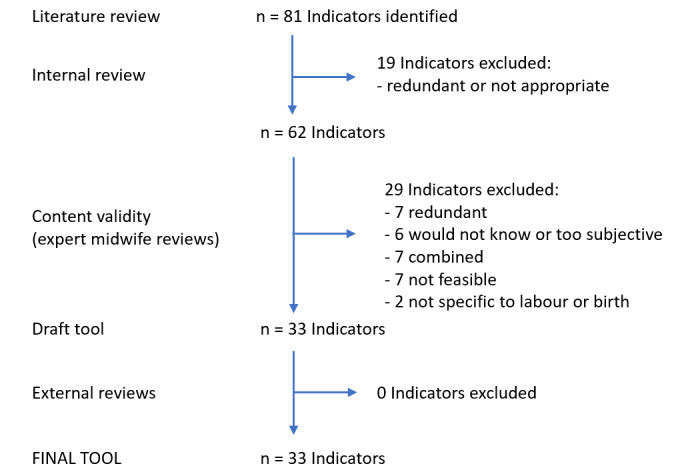
Selection of indicators for inclusion in the final maternity experience tool.

The literature review identified 18 publications meeting the pre-established criteria (**Table 1**). From these articles 81 indicators of respectful care were extracted. Of these indicators, 19 were found to be either redundant or not appropriate within a resource-constrained setting. The remaining 62 indicators were then sub-grouped under 9 domains: 1) communication/verbal interaction, 2) supportive care, 3) physical abuse, 4) non-consented care, 5) non-confidential care/lack of privacy, 6) stigma and discrimination, 7) abandonment/neglect, 8) detention/inability to pay, and 9) health facility conditions. The subsequent review of the remaining 62 indicators lead to the omission of an additional 29 indicators for the following reasons: a) redundant, b) difficult for the mother to know or highly subjective, c) combined with another indicator, d) often not feasible in a resource constrained context, and e) not specific to the labour or birth (**Figure 1**). This resulted in a 33-item perinatal experience assessment tool (**Table 2**). The final external review led to no further omissions or additions.

**Table 1:** Selected references from which potential maternity experience indicators were identified

**Article**	**Country**	**Focus**	**Assessment tool or indicators: application**
Afulani PA et al., 2018 [21]	India	Tool validation	Person-centred maternity care scale: interview of recently delivered women
Blanc AK et al., 2015 [22]	Kenya	Validity of self-reported post-partum interviews	Indicators identified through a scoping scan of published and grey literature: direct observation of deliveries and post-partum exit interviews
Stanton CK et al., 2013 [23]	Mozambique	Validity of self-reported post-partum interviews	Selected indicators based upon WHO Integrated Management of Pregnancy and Childbirth manuals [24], elements of the Mozambique humanization of birth program and events considered feasible for a woman to report: direct observation of deliveries and post-partum home interviews at 8 to 10 mo.
McCarthy KJ et al., 2016 [25]	Kenya	Validity of self-reported post-partum interviews	Indicators identified through a landscaping scan of published and grey literature: direct observation of deliveries, post-partum exit interviews and follow up interviews at 13 to 15 mo post-partum
Freeman LP et al., 2018 [26]		Validity of self-reported post-partum interviews	Indicators identified through interview with community members and leaders, health system actors: direct observation of delivery and post-partum exit interviews
Bohren MA et al., 2018 [18]	Nigeria, Guinea, Ghana, Myamar	Development and validation of direct observation and self-reported post-partum interview tools	Indicators included were identified through systematic reviews, primary qualitative research, mapping of existing tools, item consolidation, peer review by key stakeholders and piloting: post-partum home interviews (2-8 weeks)
Dey A et al., 2015 [27]	India	Adherence to clinical protocols and validity of self-reported post-partum interviews	Facility-based clinical protocols: direct observation of deliveries and 2 to 4 week post-partum interviews
Sheferaw ED et al., 2016 [28]	Ethiopia	Development of a tool	Literature review and in-depth interviews with labour and delivery clients, followed by an expert review of identified indicators: post-partum interviews within 7 weeks following delivery
Taavoni S et al., 2018 [29]	Iran	Development of a tool	Adapted from the report of a landscape analysis by the USAID-TRAction Project [5]: post-partum exit interviews
Banks KP et al., 2018 [10]	Ethiopia	Evaluation of facility-based deliveries	Adapted from Population Council Kenya tools: direct observation + post-partum exit interviews
Asefa A et al., 2015 [16]	Ethiopia	Evaluation of facility-based deliveries	Adapted from US Agency for International Development: respectful maternity care standards, USAID 2011: post-partum exit interviews
Kujawski SA et al., 2017 [30]	Tanzania	Project evaluation of facility-based deliveries	Adapted from the report of a landscape analysis by the USAID-TRAction Project [5]: post-partum exit interviews
Abuya T et al., 2015 [31]	Kenya	Project evaluation of facility-based deliveries	Adapted from Population Council Kenya tool: direct observation of deliveries + post-partum exit interviews
McMahon SA, 2014 [11]	Tanzania	Evaluation of facility-based deliveries	Indicators identified through pre-survey interviews: interviews of women who had delivered in the past 14 mo, male partners, CHWs, and community leaders
Rosen HE et al., 2015 [8]	Ethiopia, Kenya Madagascar, Tanzania, Rwanda	Evaluation of facility-based deliveries	Clinical observation of protocol checklists, including provider-client interaction: direct observation of deliveries
Sando D et al., 2016 [32]	Tanzania	Project evaluation of facility-based deliveries	Adapted from Population Council Kenya tool [33]: direct observation, immediate post-partum, and exist interviews
Sethi R et al., 2017 [34]	Malawi	Evaluation of facility-based deliveries	Adapted from USAID-TRAction Project. Exit interviews [5] and the White Ribbon Alliance’s Universal Rights of Childbearing Women Framework: direct observation of deliveries
Warren C et al., 2013 [33]	Kenya	Project evaluation of facility-based deliveries	Indicators identified through FGDs, interviews of providers and delivery observations: direct observation of deliveries and post-partum exit interviews

USAID – United States Agency for International Development, CHW – community health worker, FGD – focus group discussion

**Table 2:** Perinatal Experience Assessment Tool (PEAT) with supporting references for each indicator

Domain	Indicator	Yes*	No*	Uncertain
**Communication/verbal interaction**	During your stay did the health workers speak to you in a respectful, polite manner?	1	0	0
	Were you respectfully greeted during your admission?	1	0	0
	Did the person who assisted you in childbirth introduce him/herself?	1	0	0
	Did someone explain to you about the processes of labour and delivery?	1	0	0
	Did anyone explain to you the progress of your labour?	1	0	0
	Did you express any concerns or ask questions? If yes, were they responded to?	1	0	0
	Were you spoken to in a language you could understand?	1	0	0
	Did any health workers use aggressive, threatening language or were you shouted at, insulted or scolded during your labour or delivery?	0	1	0
**Supportive care**	Did you feel the care you received met your cultural or religious wishes/values?	1	0	0
	Were you encouraged to stand up, walk or change positions during your labour?	1	0	0
	Were you allowed to have a support person (husband, relative, friend, traditional healer) with you during labour and delivery?	1	0	0
	Were you allowed to breastfeed your baby shortly following (within one hour) delivery?	1	0	0
	Were you given your baby immediately after delivering?	1	0	0
**Physical abuse**	During your labour or delivery, were you slapped, kicked, pushed or otherwise physically hurt?	0	1	0
	Did you at any time feel you were being inappropriately touched or sexually abused?	0	1	0
	Did you feel you were in any way physically maltreated or abused during your stay (physically restrained, tied down, etc.)?	0	1	0
	Did you have a tear or cutting of your vagina during delivery that required stitching? If yes, were you given something to take the pain away? If no, leave blank.	1	0	0
**Non-consented care**	At any time, were you examined abdominally or vaginally without understanding the reasons for the exam?	0	1	0
	Was anything done to you without your understanding the reasons for it or not providing consent? Such as vaginal exam or episiotomy.	0	1	0
**Non-confidential care/lack of privacy**	Did you feel you were properly provided privacy during abdominal or vaginal examinations?	1	0	0
	When you were asked questions about your health or pregnancy was this done privately (where others could not hear)?	1	0	0
**Stigma and discrimination**	Did you feel some providers did not treat you well/respectfully because of who you are? (personal attributes: ethnicity, race, religion, SES, age, HIV status, marital status).	0	1	0
	Did you feel you were denied services because of who you are?	0	1	0
**Abandonment/neglect**	During your labour or delivery were there times when you needed help but no health worker (provider) was available?	0	1	0
	During your labour or delivery did you ask that anything not be done but it was done anyway?	0	1	0
	During your labour or delivery did you feel your wishes were neglected?	0	1	0
**Detention/inability to pay**	After your delivery were you or your baby not allowed to leave because you could not pay?	0	1	0
	Were you or your baby forced to stay against your will for any other reason than your ability to pay?	0	1	0
	At any time, were you asked to make informal payments by a health worker or other staff?	0	1	0
	During your stay, were you denied any services because you could not pay?	0	1	0
**Health facility conditions**	When you were examined was a separating partition or curtain to provide privacy provided between your bed and others?	1	0	0
	During your labour, did you have your own bed?	1	0	0
	Did you feel there were unnecessary delays in the caregiven to you?	0	1	0

SES – socioeconomic status, HIV – human immunodeficiency virus

*Add the numbers to obtain a total score.

## DISCUSSION

As stipulated in the WHO intrapartum care guidelines, every woman is entitled to be treated with dignity, provided privacy, ensured confidentiality, ensured freedom from harm and provided freedom of choice during labour and delivery [35]. The PEAT provides a user-friendly tool to measure and monitor each of these indicators of respectful maternity care. Larsen et al. [2] distinguish measures of patient experience from patient satisfaction, with the former focusing on the quality of care received and the latter on a patient’s evaluation of the care received. Patient experience measures of labour and delivery can be further differentiated based upon data collected from direct observation [8,18,27,34], post-delivery interviews [12,16,18,28,29,32] or a combination of the two[10,22-25,34]. The tool we have developed measures the quality of care and the experience of women, from their point of view, but does not ask about satisfaction of the care received.

Interviews can be further characterized by the timing and location of the interview (postpartum interviews at the time of discharge [20,28] vs post-discharge home interviews [18]), with each having distinct advantages and disadvantages. The former has logistical and cost efficiencies while home interviews may be less vulnerable to reporting adverse experiences. We have considered the cost-savings and ease of administering the interview in the facility rather than during the postpartum period at home. There are some advantages and disadvantages to administering the survey to women in the facility rather than in the comfort of their home. In the facility there might be a perception that they would not be given good treatment (appropriate medications or monitoring at discharge) if they divulge gaps or negative aspects in their care. Kruk et al. [17] found a possible courtesy bias when interviewing postpartum women about experiences of disrespectful treatment in the facility (19% reported abuse) as compared to re-interviewing the same women in their homes (28% reported abuse). This can be mitigated through the survey being done by other persons who are not involved in her care. The advantage to a facility-based survey is that it saves costs and is much easier to collect and store the data. Monitoring and supervision are more easily available at the facility. The PEAT is a patient experience measure, preferably administered at the time of discharge to be offered in a personal interview format.

Bohren et al. [15] completed a large comprehensive mixed-methods systematic review of mistreatment of women and developed a typology of domains of disrespect to be used in further research and for tool development. Our study and its tool, although modest, was reviewed and modified by midwives who had worked in over a dozen countries. We offer the survey as a pragmatic way to monitor quality of care, and to determine areas that can be improved.

The PEAT is intended to be used to: 1) evaluate the quality of perinatal care received, 2) allow for a focus on specific domains of quality care, 3) facilitate structural, procedural and health provider behavioural improvements, and 4) provide evidence-based data guiding perinatal health policies and their monitoring. PEAT scores are context specific, with findings to be interpreted within the contexts of available human and material resources, patient characteristics such as ethnicity, religion or wealth, and the presence of systemic racism, and facility cultures. These will need to be taken into consideration and treated with caution when comparing experiences among women in different facilities.

The tool is intended to be completed by independent, trained, and supervised interviewers. Preferably, women who have recently given birth would be interviewed at the time of their discharge from a delivery facility. Alternatively, a home interview could be conducted during the postpartum period. It is anticipated the interview will require, on average, 15 to 20 minutes to complete. Definitions of terminology used in the tool are listed in **Table 3**. For each indicator, expressed as a question, the interviewer will indicate “yes”, “no” or “uncertain”. The decision to limit responses to “yes”, “no” or “uncertain” was taken to reduce subjectivity, an inherent challenge with interviews dependent on client recall.

**Table 3:** Definitions of terminology used in the Perinatal Experience Assessment Tool (PEAT)

Term	Definition
Aggressive language	Speaking in an angry or violent way towards the mother
Cultural wishes	Customary or traditional practices of the mother’s ethnic group or otherwise defined population the mother is part of
Episiotomy	A surgical cut made at the opening of the vagina during childbirth, to aid a difficult delivery and prevent rupture of a mother’s tissues
Ethnicity	A mother’s social group that shares a common and distinctive culture, religion, language, or other distinguishing characteristics
Informal payments	Additional payments on top of any formal payments, sometimes considered illegal
Inappropriate touch	Unwanted touching that is perceived by the mother to be sexual in intent
Religious wishes	Behaviours or practices that are dictated by or consistent with the mother’s religious beliefs
Sexual abuse	Any unwanted sexual act forced upon the mother, whether verbal or physical. If a respondent reports sexual abuse, she should be asked permission to report this.
Race	Any group the mother belongs to based upon physical traits regarded as common among people of a shared ancestry
Support person	A husband, relative or friend who assists the mother with physical needs and/or provides psychological reassurance during labour or delivery

Referring to **Table 2**, there is a one (1) in the column under the appropriate “yes” or “no” response indicating respectful care. To score, the numbers are counted. While higher attainable scores are desired, no optimal threshold score is being recommended at this time.

Aggregated scores can then be used to situate a maternity care service at one point in time and monitor trends over time. Scores will vary by the availability of human and material resources found in a facility. Shortages of qualified human resources may reduce the quality of care provided to child-bearing women.

Strengths of this tool include its ease of use, relative cost efficiency and inclusion of indicators from a wide range of published measurement tools. The survey can be administered within a short amount of time by personnel who have been trained but are not necessarily health care workers. There is no need to observe and record in real time the experiences of service users at the facility, which would interfere with privacy and possibly a positive birth experience. In areas with good internet connectivity and high use of mobile devices, the survey could be completed online by service users.

The tool has limitations. It may not capture all aspects of the quality of birthing individuals’ experience. The recommendation to complete the interview while at a health facility has the potential to create a reluctance among respondents to fully report negative experiences. This will be diminished by employing interviewers independent of the facility. Using the tool successfully will require training, supervision and monitoring of interviewers and a committee who meet to review the interview results and plan for quality care improvement. An additional limitation is the need for further criterion validity field testing of the tool among populations of varied cultural and socioeconomic backgrounds.

## CONCLUSIONS

The PEAT has been developed for use in low-resource countries as a quality assurance tool for maternity health facilities. The tool can be applied by an independent trained auxiliary or registered health care worker or an independent interviewer, preferably at the time of discharge of women from the facility to provide a measure of respectful care. Results can then be used to improve the quality of care on maternity wards through in-service training of staff, changes in infrastructure, modification of labour and delivery communication and processes and strengthened policies.
